# The Influence of Social Media Usage and Perceived Government Market Orientation on Travel Intention to an Internet Celebrity City: Exploring the Mediating Effects of Place Attachment and Perceived Value

**DOI:** 10.3390/bs14080694

**Published:** 2024-08-09

**Authors:** Wu Tang, Cevat Tosun, Ahmad Edwin Mohamed, Sinan Uslu

**Affiliations:** 1 School of Tourism, Hospitality & Event Management, Universiti Utara Malaysia, Sintok 06010, Malaysia; tang_wu@gsgsg.uum.edu.my (W.T.); edwin@uum.edu.my (A.E.M.); 2 School of Business, The George Washington University, Washington, DC 20052, USA; 3 Faculty of Tourism, Necmettin Erbakan University, Konya 42090, Turkey; suslu@erbakan.edu.tr

**Keywords:** social media usage, perceived government market orientation, place attachment, perceived value, travel intention, internet celebrity city

## Abstract

As the usage of social media continues to grow and local governments expand their promotional efforts, more internet celebrity cities (ICCs) are emerging as popular tourist destinations. To investigate the internal mechanisms that affect tourists’ travel intentions to such destinations, place attachment and perceived value were proposed as two factors reflecting their deeper emotional connections and practical assessments to visit ICCs. This study was conducted to examine the relationship between social media usage, perceived government market orientation (PGMO), place attachment, perceived value, and travel intention in the context of Chongqing, a popular ICC in China. To evaluate the research hypotheses, 750 valid questionnaires were collected and analyzed by structural equation modeling. The results showed that social media usage had a positive impact on tourists’ travel intention to Chongqing, while PGMO did not significantly influence travel intention. However, both place attachment and perceived value had mediating effects in the relationship between social media usage, PGMO, and travel intention. This paper concludes with theoretical implications, suggestions for further research, and recommendations for practice.

## 1. Introduction

Social media has had a revolutionary impact on the development of the tourism industry [1]. Social media platforms such as Instagram, Facebook, and TikTok have become integral sources of information and inspiration for tourists [2]. They allow travelers to explore destinations virtually through user-generated content, influencers’ recommendations, and official tourism promotions [1,3]. Cities and destinations have adapted to this trend by actively using social media as a primary tool for marketing. They create engaging content that showcases their unique attractions, cultural experiences, and local flavors to capture the interest of potential visitors [4]. By strategically leveraging social media platforms, destinations can significantly enhance their visibility, cultivate a positive image, and ultimately attract more tourists [5].

In China, social media platforms like Sina Weibo (Chinese Twitter) and Douyin (Chinese TikTok) have significantly promoted cities such as Chongqing, Beijing, and Hangzhou. This has resulted in soaring city brand awareness and influence over a short period. As a result, these cities have quickly become popular internet celebrity destinations. Postma, Buda [6] emphasized that the spread of social media had reinvigorated the growth of city tourism. According to Shirly [7], Chongqing, known as an 8D mountain city, ranked first among the top 100 cities in China for tourism influence and won the “2022 City Tourism Online Popularity Award”. Over the past few years, it has become one of the hottest internet celebrity cities (ICCs).

According to Wang [8], the popularity of social media is a precondition for the emergence of ICCs. However, another important factor is the force behind the scenes such as local governments. Since city tourism can bring significant growth to the local economy, Chen, Li [9] implied that the local government had a strong motivation to transform cities into internet celebrity tourist destinations through market-oriented initiatives. Taking the city of Ganzi in China’s Sichuan province as an example, the director of the city’s Cultural and Tourism Bureau successfully transformed Ganzi into an internet celebrity tourist destination by posting short cosplay videos on social media in 2022, which resulted in a large number of tourists [10].

Scholars have investigated the influence of social media usage (SMU) [11,12,13] and perceived government market orientation (PGMO) on tourists’ travel intentions (TI) [14,15]. However, there has been little research on the internal mechanisms that affect tourists’ intentions to visit a tourist destination. Yang [16] implied that potential tourists’ intentions to visit internet celebrity tourist destinations stemmed to some extent from an emotional and psychological longing for these destinations, whether through curiosity, novelty, or attachment. In the prevalent era of social media, tourists’ use of social media often aids in fostering their sense of place attachment (PA) [17]; furthermore, the marketing efforts of destination governments frequently play a facilitating role in the formation of tourists’ PA [16]. Thus, place attachment (PA) was examined as a mediator in this study.

Nevertheless, beyond internet fame, factors such as the quality of city tourism management, infrastructure, service, and value for money also significantly impact tourists’ intentions to travel when choosing among many tourist destinations [18]. When they perceive all of these aspects of an ICC either through social media usage or government marketing orientation, the perceived value (PV) of the destination is established [19,20]. Accordingly, PV was examined as another mediator in this study.

Therefore, the objective of this research was to identify the relationships among SMU, PGMO, PA, PV, and Chinese tourists’ TI to the ICC of Chongqing. Particularly, this study aimed to determine the mediating effects of PA and PV in these relationships. The results of this study can enrich the theory of PA and PV. The findings can also benefit city governments in developing a market-oriented government in tourism and provide suggestions to destination tourism organizations during their social media marketing activities.

## 2. Literature Review

### 2.1. Internet Celebrity City and Social Media Usage

Among the groups that have developed since the rise of social media, “Internet celebrities” have been one of the most distinctive; they have been frequently discussed in China since 2015 [21]. Internet celebrities refer to people who have become famous on social media and online communities because of certain behaviors or events that are noticed by the general public [22,23]. With the prevalence of social media, internet celebrities have extended beyond individual people to encompass physical locations and ideas or symbols. Accordingly, internet celebrity attractions, cities, restaurants, etc., have emerged [24]. Lei [25] characterized ICCs as cities that became popular in a short period of time in the context of media convergence, resulting in an increase in traffic online and visitors offline.

According to Yang [16], the rise of ICCs can be attributed to three primary factors. Firstly, the rapid advancement of internet technology has led to the emergence of various new social media platforms, which have transformed people’s habits of acquiring information. Secondly, user-generated content, encompassing text, images, videos, and reviews posted by users on the internet, provides diverse and personalized content that is convenient for travelers. Thirdly, the inherent characteristics of ICCs, such as their unique historical culture, local music, cuisine, scenic landscapes, technological infrastructure, and regional dialects, contribute significantly to their popularity on the internet. Wang [8] further explained that the rise of social media was crucial for the emergence of ICCs, while government market-oriented efforts in tourism served as the driving force behind it. Furthermore, city tourism contributes significantly to urban economic growth by driving development in key sectors like lodging, transport, and restaurant chains [6]. City governments are entrusted with the fundamental task of cultivating distinctive tourism attributes and enhancing competitiveness [26].

Meanwhile, the usage of social media has become a prevalent online activity. In 2021, the number of global social media users exceeded 4.26 billion individuals, with a projected increase to 6 billion by 2027 [27]. People engage with social media for a multitude of purposes, ranging from entertainment and communication to information searching [28]. Swar and Hameed [29] described social media usage (SMU) as the actions taken by individuals to exchange information, express viewpoints, and share interests through interactive online platforms and tools. In the field of tourism, travelers often utilize social media to search for travel information before embarking on their journey [30].

For tourism providers, public organizations, such as destination-marketing organizations, view social media as a crucial tool for promoting destinations. Islam [4] implied that social media offered them a chance for extensive exposure and rapid exchange of diverse and integrated information. Song and Schuett [17] advocated that marketers should endeavor to formulate suitable social media strategies to foster PA between virtual visitors and their tourist destinations. Furthermore, an increasing number of studies have found that the usage of social media can effectively influence consumers’ PV, making it an important marketing tool for businesses [20,31,32], since social media users are directly impacted by both the costs and benefits in their search for travel information [2].

### 2.2. Perceived Government Market Orientation

The government is an essential player in tourism development, with varied degrees of involvement depending on the stage of tourism development [26]. In contrast to tourism in developed countries like the United States, China’s tourism industry has a fairly young history. However, the tourism authorities in China exercise comprehensive control and actively participate in various aspects of tourism development, including travel service management, transportation coordination, and destination promotion [33].

Governments must adopt market-oriented behaviors or measures, similar to the private sector, to assist in the development of tourism [34]. Market orientation, as a research construct, has various definitions, but there is a common perspective that it is related to information processing and is aimed at implementing marketing concepts to generate value and competitiveness [35]. Parra-Lopez, Martínez-Gonzalez [36] suggested that market orientation was the behavior of organizations in formulating market intelligence and policies to meet consumer needs and expectations, and this behavior was ultimately perceived by consumers. Kamarulzaman, Khairuddin [37] further divided market orientation into three subconstructs: consumer orientation, competitor orientation, and internal coordination within the organization. In the tourism sector, Zhang [14] suggested that government market orientation could be characterized as a series of market-oriented initiatives taken by the government to manage the overall image of a tourist destination, the overall regional tourism environment, and tourism products. These behaviors aimed to satisfy the requirements and expectations of tourists, ultimately seeking to be recognized and appreciated by them.

According to Song, Jiang [38], the marketing performance of the government, which acts as one of the most important entities of destination-marketing organizations, has gradually attracted the attention of scholars in tourism since the 1990s and has been further developed by scholars such as Faulkner [39] and Pike [40]. The city government adopts various marketing strategies to develop urban tourism, including city branding and promotion [41], event marketing [42], sustainable development and eco-tourism [43], tourism service and experience enhancement [44], and more. Additionally, Cervi, Calvo [45] investigated how the new digital marketing strategies for city branding were implemented during COVID-19 in the 26 most visited cities, which was found to play a promoting role in city tourism. Zhang [14] stated that market-oriented efforts by the government were beneficial for the destination’s image and reputation and tourists’ intentions to visit. Furthermore, Zhao and Dou [15] indicated that the proactive marketing efforts by local governments, including marketing promotion, consumer rights protection, and tourism information management, had significantly improved destination image and contributed to the increased influx of tourists.

In the current era dominated by social media, the government’s market-oriented approach is more perceptible to prospective tourists. Within tourism studies, scholarly research on government marketing predominantly concentrates on its significance [46], the evaluation of government marketing effectiveness [38], and deficiencies in government marketing endeavors [47]. Although a few scholars have investigated the influence of PGMO on travel intentions, these studies did not emphasize the subject of ICCs in social media contexts [14]. This study aims to address this gap.

### 2.3. Place Attachment

The concept of PA originated in the field of environmental psychology and was introduced to the tourism literature during the 1980s [48]. Despite lacking a robust theoretical foundation, PA is significant in effectively marketing destinations, as it correlates with tourist decision-making behavior [49]. Morgan [50] suggested that PA could be construed as the emotional or functional connection between an individual and a specific geographical locale, encompassing the perceived significance of this connection. In essence, tourists develop a profound emotional or utilitarian bond with a destination based on their cumulative experiences. Several studies in the tourism sector have delineated two key components of PA: place identity and place dependence [51,52,53,54]. Place identity encompasses the symbolic or emotional connections to a specific location, whereas place dependence refers to the practical connections that highlight the significance of a place in meeting one’s functional objectives or activities [55].

Researchers have studied the influence of PA on revisiting destinations where one has lived before [52]. A study on visitors to the revitalization park in Jakarta found that PA significantly influenced tourists’ intention to revisit [54]. Similarly, Stylos, Bellou [56] stated that PA plays a role in moderating the relationship between destination image and revisit intention.

It is widely believed that attachment to a place is gradually formed through frequent and long-term interactions [52]. However, emerging studies have indicated that individuals have the capacity to develop emotional bonds with unfamiliar locations, fostering a unique and personal connection with these places [57,58]. In the era of social media, tourists often acquire information about travel destinations through short videos, images, and other forms of content. They can easily immerse themselves in these materials, often developing a sense of presence and forming special emotional connections and dependencies as a result [57]. Therefore, more research is needed to examine tourists who have not visited a location but can still develop a sense of PA to tourist destinations.

### 2.4. Perceived Value

PV is one of the most studied variables in marketing literature, but academics have not yet reached a consensus on its definition [18]. According to Zeithaml [59], PV refers to how customers evaluate a product’s overall usefulness based on what they receive and what they relinquish. Most researchers have suggested that PV is a multidimensional construct [32,60]. In tourism, researchers have argued that the tourism experience encompasses various attributes, and unidimensional measurement overlooks the emotional, situational, and extrinsic attributes of this experience. Williams and Soutar [61] proposed that PV included functional value, social value, economic value, emotional value, and novelty value. They also found that all dimensions had a positive impact on tourist satisfaction. PV in this study comprises three components: novelty value, economic value, and functional value.

PV has long been recognized as one of the most significant determinants of tourist behavior and intentions. Caber, Albayrak [18], in a study of young tourists’ intentions to travel in Turkey, found that the functional value of PV was the strongest predictor of young tourists’ visits to destinations. Lee, Yoon [62] explained that PV had a significant impact on satisfaction, thus influencing tourists’ decision to recommend the destination to others.

Under the context of ICCs, some scholars from China have attempted to explore the internal mechanisms of tourists’ intentions to visit internet celebrity destinations from psychological perspectives, such as the empathy process, yet a unified understanding has yet to be established [63]. Therefore, this study introduced PA and PV as mediating variables for investigation, aiming to deepen the understanding of the psychological mechanisms of potential tourists, thereby contributing to the field’s research.

## 3. Hypothesis Development

ICCs have much higher social media exposure and coverage compared to other cities. Therefore, tourists with higher SMU are often exposed to more travel information about specific cities through social media, which in turn enhances their intentions to visit these cities [24]. Meanwhile, the market-oriented behavior of local governments serves as a significant driver behind the emergence of ICCs [8] and has been found to directly influence tourists’ TI [14]. Therefore, hypotheses H1 and H2 are presented as follows:

**H1:**  *SMU has a direct positive effect on TI.*

**H2:**  *PGMO has a direct positive effect on TI.*

Meanwhile, the role of SMU in influencing PA has been verified by many studies. For example, Hosany, Buzova [52] surveyed 410 potential Spanish tourists and found that tourists develop PA and visit intention towards destinations they have not traveled to before. A study on Pakistanis who watched tourism destination videos on social media discovered that advertisement value had a positive effect on intentions to travel, with PA serving as a mediator [64].

In addition, the social media environment encourages user interaction and engagement. When users browse websites, they are often influenced by the interactive and informational content about specific products on the website, thereby affecting consumers’ PV of the product [65]. For example, a recent study found that, even if they did not make purchases, consumers could still be influenced by Instagram advertisements in terms of their PV [32]. Simultaneously, a study of 502 social media users revealed that social media marketing activities can also significantly impact consumers’ PV [20]. Accordingly, hypotheses H3 and H4 are presented as follows:

**H3:**  *SMU has a positive impact on PA.*

**H4:**  *SMU has a positive impact on PV.*

According to Yang [16], cultural promotion is a crucial component of city tourism promotions undertaken by city governments. They aim to cultivate an emotional connection among tourists with destination cities. Zhu and Wang [19] explained that the governments’ tourism marketing efforts often allow tourists to perceive cost-effectiveness in aspects such as the destination city’s tourism infrastructure, quality assurance of urban services, and price control. Accordingly, this study posits that local governments actively engage in activities such as shaping the city’s image and conducting tourism marketing in their efforts to promote city tourism. Simultaneously, they proactively meet the needs and expectations of tourists through a series of market-oriented measures [15]. When exposed through media promotion and eventually perceived by tourists, these measures are expected to impact tourists’ PA and PV. Hence, the following hypotheses are proposed:

**H5:**  *PGMO has a positive impact on PA.*

**H6:**  *PGMO has a positive impact on PV.*

Behavioral intention pertains to an individual’s propensity to take action toward a particular activity or object [66]. TI, as a crucial outcome variable, refers to the likelihood or attitude inclination of tourists towards visiting a particular destination [67]. Specifically, destination selection intention in tourism research involves the attitude inclination of potential tourists towards whether they intend to visit a particular tourist destination. This inclination is influenced by factors such as the appeal of the destination and other environmental factors [68].

A considerable amount of research has indicated that PA [42,51] and PV [69,70] significantly impact TI. According to Jeong, Yu [42], PA positively influences the TI of South Korean tourists towards sporting events. Al-Adamat, Al-Gasawneh [69] stated that PV positively influences the TI of potential Saudi visitors to Jordan. Therefore, this study proposes hypotheses H7 and H8, as follows:

**H7:**  *PA has a positive impact on TI.*

**H8:**  *PV has a positive impact on TI.*

Moreover, PA, as a mediating variable, has been widely studied. For example, in a study of Pakistanis watching tourism destination videos on social media, it was found that PA serves as a mediating variable between the value of social media advertising and TI [64]. Nursyamsiah and Setiawan [54] pointed out that PA mediates the relationship between tourist satisfaction and the intention to revisit. Hence, this study proposes hypotheses H9 and H10 as follows:

**H9:**  *PA mediates the relationship between SMU and TI.*

**H10:**  *PA mediates the relationship between PGMO and TI.*

Social media marketing activities have been proven to influence PV, subsequently impacting consumer satisfaction, with PV acting as a mediating factor [20]. When Muslim tourists choose to travel to non-Islamic countries, the destination’s Muslim-friendly traits positively shape the PV, thereby influencing their TI [71]. The following hypotheses, H11 and H12, are proposed accordingly:

**H11:**  *PV mediates the relationship between SMU and TI.*

**H12:**  *PV mediates the relationship between PGMO and TI.*

**Figure 1:**
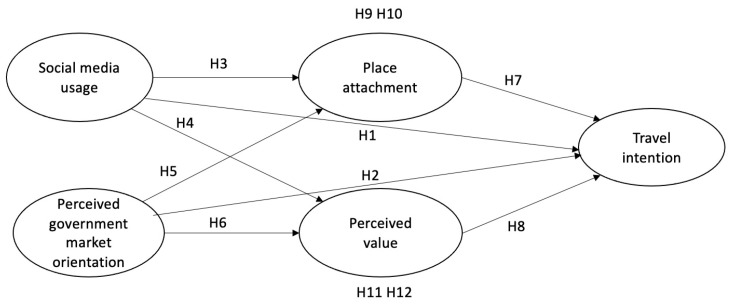
Proposed model.

Figure 1 shows the research framework. This study sought to investigate the interconnections between SMU, PGMO, PA, PV, and TI to one of the hottest Chinese ICCs: Chongqing.

## 4. Methodology

The study was crafted as an empirical study, aimed at investigating the factors that influence tourists’ intentions to visit an ICC. Given that this study focused on social media users, a large and diverse population, quantitative research was chosen as the appropriate methodology since it can extend its findings to larger populations by employing a significant number of participants to address the research questions [72].

### 4.1. Study Setting

As one of the earliest and most popular ICCs, Chongqing was selected as the context for this study. In 2017, a Doyin user uploaded a short video about Chongqing, named “Train goes through a 19-storey block of flats in Chongqing”, which received 469,000 likes and 17,000 shares [73]. In the same year, short videos about Chongqing were played 11.36 billion times, giving the city the highest rank among cities from China [74]. In addition, according to recent statistics [75], the number of visitors to Chongqing ranked first among all the cities in China for five consecutive years from 2017 to 2021.

Meanwhile, the government’s market-oriented initiatives in Chongqing have been widely recognized in China. In a recent annual selection of city branding cases, the Chongqing government received the national city marketing award for “The city that favors tourists the most in 2022–2023” [76]. Based on this discussion, as well as the first author’s sociocultural proximity and personal experience with this city, Chongqing as a tourist destination and ICC appeared to be an excellent unit of analysis to study the relationship between SMU, PGMO, PA, PV, and TI.

### 4.2. Data Collection and Sampling

Determining an adequate sample size that is representative is crucial for ensuring the reliability of research findings [77]. Krejcie and Morgan [78] suggested that the sample size should be more than 384. Comrey and Lee [79] recommended a sample size of 300 as good, 500 as highly satisfactory, and 1000 as outstanding. Therefore, an appropriate sample size for this study was determined to be greater than 500. On the other hand, Despite the lack of unanimous agreement regarding the ideal sample size for SEM, a pivotal sample size of 200 has been suggested as appropriate for conducting analysis and testing models [80,81]. Consequently, any number exceeding 200 is deemed sufficient to establish a solid statistical framework for data analysis [82].

Before data collection, a cohort of 50 participants was purposively chosen from the target population to pilot the survey via the social media site Douyin. Subsequently, a reliability examination was conducted to evaluate the utility of the scale. It was observed that all composite reliability values (SMU, 0.875; PGMO, 0.912; PA, 0.892; PV, 0.924; TI, 0.949) surpassed the threshold of 0.70 [83], indicating the reliability of the scale. Since one objective of this study was to investigate the effects of PGMO, this study distributed online questionnaires through the account of the Chongqing Culture and Tourism Commission in Sina Weibo and Douyin. First, we chose a city promotion post by the Chongqing government with the most page views. Then, 1000 questionnaire invitations were distributed to the commentators by short messages in Sina Weibo and Douyin through convenience sampling. Questionnaires were self-administered, and only those who had not previously visited Chongqing could answer the questionnaire. In the end, 762 questionnaires were collected; after removing 12 invalid ones, 750 valid questionnaires were used for analysis.

### 4.3. Measures

The measurement items for each construct in the hypothesized model were derived from validated measurements utilized in prior research. To assess each construct, rating scales with 7 points on a Likert scale were used (1 = Strongly Disagree, 7 = Strongly Agree). The concept of SMU was refined from Ellison, Steinfield [84], and the concept of PGMO was adapted and modified from Parra-Lopez, Martínez-Gonzalez [36] and Gray, Matear [85]. Meanwhile, the scales for the remaining constructs were determined by incorporating the existing literature—PA [42,86], PV [61,87,88], and TI [89]. Back translation between Chinese and English was employed to ensure consistency in conveying the same information in both versions of the questionnaire [90]. The questions for all measures are displayed in Table 1.

### 4.4. Data Analysis

This study employed structural equation modeling (SEM) to conduct data analysis using Statistical Package for the Social Sciences 20 (SPSS) and Analysis of Moment Structures 24 (AMOS). As explained by Kline [91], AMOS is frequently utilized in quantitative empirical research due to its strong capabilities in SEM, allowing researchers to examine both direct and mediating effects among variables. This tool facilitates a systematic investigation of direct and indirect interactions through intermediate pathways, thereby advancing the understanding of the underlying mechanisms impacting research outcomes. In addition, AMOS employs Maximum Likelihood estimation for SEM, making it a valuable tool for validating the researched model [83]. Accordingly, the data analysis in this study was divided into three stages: descriptive statistics analysis, measurement model validation, and structural model analysis. First, frequency and percentage distributions of basic sample information, as well as the mean and standard deviation of results, were analyzed. Then, following the views of Anderson and Gerbing [92], confirmatory factor analysis was utilized to test the measurement model to confirm the convergent and discriminant validity of constructs. Subsequently, the structural model was validated for its model fit, followed by path analysis and mediation effect examination to verify the research hypotheses of this study.

## 5. Findings

### 5.1. Demographic Findings

Among the 750 participants in the research, more respondents were female (n = 520, 69.3%). Most participants were in their 20s (n = 421, 56.1%). The main monthly income was below 5000 Yuan (n = 383, 51.1%). As for education level, most had a bachelor’s degree (n = 509, 67.9%). A previous study found that females dedicated a greater amount of time weekly to the Internet and displayed more favorable attitudes toward both online and offline sources of travel information [93]. Meanwhile, recent consumption data statistics from Douyin [94] show that females account for over 70% of cultural and tourism consumption on the platform. Moreover, females constitute more than 66% of the users in the Chinese main social media platforms such as Douyin and Xiaohongshu, with a predominance of young people [95,96]. Therefore, the above sample data aligned with the actual demographic characteristics of Chinese social media users.

On the basis of the sample statistics, the mean of all variables ranged from 4.670 to 5.940, and the standard deviation varied between 1.188 and 1.571. Meanwhile, the skewness value fell into a range of −1.1911 to −0.566, while the kurtosis value spanned from −0.287 to 4.174. According to the criteria outlined by [91], the skewness value should be within ±2, and the value of kurtosis should be within ±7. Therefore, this study’s results can be considered consistent with a normal distribution and suitable for further analysis.

### 5.2. Measurement Model

Kline [97] suggested that factor loadings should ideally surpass 0.6, with values above 0.7 considered optimal. For a reliable measurement, the composite reliability must exceed 0.7 [83], while the average variance extracted (AVE) should be greater than 0.5 [98]. For the variables in this study, factor loadings ranged from 0.677 to 0.942; composite reliability ranged from 0.920 and 0.960; and the AVE ranged from 0.661 to 0.865. These statistics suggested that this study had strong convergent validity [83,99] (Table 2).

This study examined discriminant validity using the rigorous AVE method. According to Fornell and Larcker [98], discriminant validity should take into account both convergent validity and the relationships between constructs. Thus, each construct’s square root of AVE should exceed its correlation coefficients [100]. As shown in Table 3, the diagonal square roots of AVE for each construct were greater than the correlation coefficients outside the diagonal. Discriminant validity was acceptable.

### 5.3. Structural Model and Hypothesis Testing

**Figure 2:**
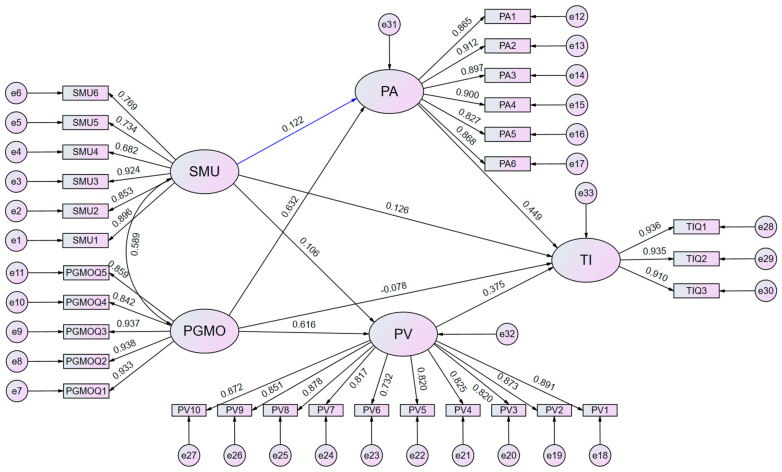
Structural equation modeling results.

Following the application of a maximum likelihood estimation approach, the structural model exhibited a favorable level of fit. The structural model’s overall goodness-of-fit indices satisfied the specified thresholds, as indicated here: χ^2^ = 706.340, χ^2^/df = 1.775, goodness of fit index = 0.973, adjusted goodness of fit index = 0.968, relative fit index = 0.971, comparative fit index = 0.988, normed fit index = 0.973, incremental fit index = 0.988, root mean square error of approximation = 0.032, and nonnormed fit index (Tucker–Lewis index) = 0.987 [101,102]. The relationships are shown in Figure 2.

#### 5.3.1. Direct Relationship Testing

Direct relationships were examined first. As shown in Table 4, SMU had a positive influence on TI, PA, and PV. However, PGMO did not have a positive impact on TI. In addition, PGMO had a significant impact on PA and PV. Both PA and PV had positive impacts on TI. Thus, all the hypotheses of direct relationships were supported except H2.

The findings revealed that SMU positively impacted TI, PA, and PV to the ICC as a tourist destination, aligning with findings from previous studies by Wu [24], Hosany, Buzova [52], Keng and Ting [65], and Kim, Chung [32]. Viewing videos or images of ICCs on social media and engaging in immersive online browsing can help arouse the intention to travel [21], can make tourists feel as if they are physically present, and can evoke emotional resonance [103]. This can ultimately lead to the development of a strong attachment to the place. Through extensive exposure on social media, tourists perceive unique urban landscapes, tourism pricing, and the level of tourism management related to ICCs. Driven by the interactive features of social media, tourists often develop a sense of PV for the destination even before their journey begins.

Interestingly, the results showed that the influence of PGMO on tourists’ PA and PV was relatively substantial compared to SMU. Social media users often encounter various information distractions, not solely focusing on short videos and images related to tourism [104]. However, despite the increasing involvement of Chinese local governments in the tourism industry, the market-oriented actions taken by local governments still provide a refreshing perspective for tourists. Furthermore, government market-oriented actions are purposeful, and the government’s related initiatives are closely related to tourists’ PA and PV [19].

However, this study found that PGMO did not have a positive impact on TI, which was inconsistent with the findings of Zhang [14]. On the contrary, PGMO positively impacted tourists’ PA and PV to the destination, consistent with recent studies by Yang [16] and Zhao and Dou [15]. It can be observed that if tourists perceive that the government’s market orientation is unrelated to PA and PV, their intentions to travel will not be affected. As a socialist country, the Chinese government inherently possesses authority, making it easily trusted. When local governments implement positive market-oriented initiatives that are perceived by tourists, these tourists are likely to develop favorable feelings and trust towards the tourist destinations. This occurs even before the tourists visit, creating a situation where PA is formed. Additionally, local governments’ market-oriented behaviors encompass destination image promotion, regulation of tourism business prices and services, and initiatives facilitating tourists’ travel [105]. However, if tourists cannot perceive these initiatives emotionally or in terms of benefits, they will not develop TI.

#### 5.3.2. Mediating Effects Testing

According to MacKinnon, Lockwood [106], the bootstrap method has been demonstrated to have greater statistical power in examining mediating effects than causal steps and the product of coefficients methods. Moreover, the resampling methods provide more accurate results compared to single-sample tests. Therefore, the bootstrap method was utilized in this study. As shown in Table 5, the confidence interval of all four indirect relationships did not include 0, indicating that these mediating relationships were established. Therefore, H9, H10, H11, and H12 were supported.

Based on the analysis findings, both PA and PV were found to mediate the relationship between SMU and PGMO. SMU and PGMO serve as stimuli affecting tourists’ TI, but what truly influences TI are tourists’ PA and PV. In other words, the underlying factors of PA and PV carry more weight in determining whether individuals actually have the intentions to visit a destination. These factors reflect deeper emotional connections and practical assessments that directly impact tourists’ intention to travel.

## 6. Conclusions and Discussion

### 6.1. Theoretical Implications

This research adds to the limited body of literature investigating the ICC of Chongqing as a potential travel destination, uniquely incorporating analyses of both SMU and PGMO within this specific context. First, the existing literature on PGMO is scant and predominantly qualitative in approach. This study employed quantitative research methods to investigate the impact of PGMO on tourists’ TI, thereby addressing deficiencies in the current research landscape.

Second, research on tourists’ PA and PV has traditionally focused on tourists’ perceptions of destinations after their travels, influencing their intention to revisit. However, this study revealed that tourists could develop PA and PV even without physically visiting these places, which in turn influences their intention to travel. This finding is a rich supplement to the theories of PA and PV.

Finally, and most importantly, tourists flocking to ICCs are driven by the prevalence of social media and local governments as driving forces behind. However, there is limited research on the intrinsic mechanisms shaping tourists’ travel intentions. This study addresses a gap by evaluating the mediating effects of PA and PV, which is a major innovation in this research.

### 6.2. Practical Implications

The findings offer practical implications. First, governments should emphasize PA and PV in city promotions. When promoting destination tourism, local governments should avoid generic and unremarkable advertising. Marketing efforts can focus on content related to PA, highlighting local tourism characteristics that meet the needs of tourists, bridging the gap between tourists and the city and enhancing trust and favorable impressions of the city. Meanwhile, as crucial stakeholders in city tourism, local governments can strive to improve city management, control tourism pricing, optimize transportation conditions, enhance the quality of tourist services, and strengthen tourism training for local businesses. By emphasizing these aspects in promotional activities, local governments can enhance tourists’ PV, attracting them to travel to the city.

Second, before traveling, tourists not only use social media to gather information about tourist attractions, food maps, and culture but also rely on various evaluations of the destination from travel bloggers and past visitors [2]. Accordingly, the electronic word of mouth for city tourism should receive special attention. Negative evaluations undoubtedly affect tourists’ TI [107]. Therefore, local governments, destination tourism organizations, and tourism service providers can establish specialized departments or assign individuals to closely monitor relevant online evaluations. In the case of negative reviews, proactive measures should be taken to mitigate or dispel their negative impact on the destination. For positive reviews, active promotion can be undertaken to leverage their positive influence, attracting tourists to visit the city [70].

### 6.3. Study Limitations and Future Development

This study employed a questionnaire distributed through social media platforms for convenience sampling. The sample primarily consisted of women (69.3%), who may be more susceptible to the influence of PA. Additionally, a majority of the respondents in this study had relatively high educational qualifications, with 92.9% holding a diploma or degree, and 56.1% were in their 20s. Higher educational levels and younger age groups are often associated with increased frequency of SMU, potentially resulting in an incomplete representation of attitudes and intentions among those with lower educational qualifications or in different age groups.

For future research, it may be beneficial to target those with lower educational qualifications and relatively older age groups. Furthermore, there is currently limited research on the TI of tourists towards ICCs. TI is often influenced by multiple factors. Subsequent investigations could explore supplementary variables, such as social media influencers and destination image.

## Figures and Tables

**Table 1:** Measurements of variables.

Code No.	SMU
SMUQ1	Social media is part of my daily activities.
SMUQ2	I proudly tell others that I am using social media.
SMUQ3	Social media has become an integral aspect of my everyday routine.
SMUQ4	If I don’t log in to social media for a while, I feel disconnected.
SMUQ5	I feel like I am a part of the social media community.
SMUQ6	If social media were to shut down, I would feel regretful.
Code No.	PGMO
PGMOQ1	The tourism policies of the Chongqing government consider the practical needs of tourists.
PGMOQ2	The tourism agencies of the Chongqing government consider the future expectations of tourists.
PGMOQ3	The tourism agencies of the Chongqing government consider the changing circumstances of tourists.
PGMOQ4	In shaping the city’s tourism image, the Chongqing government does a better job than other cities.
PGMOQ5	In conducting tourism marketing activities, the Chongqing government does a better job than other cities.
Code No.	PA
PAQ1	Chongqing is my top choice for travel.
PAQ2	I prefer Chongqing over other cities.
PAQ3	The feeling Chongqing gives me is incomparable to anywhere else.
PAQ4	Traveling to Chongqing holds great significance for me.
PAQ5	Chongqing is a very special city to me.
PAQ6	I strongly endorse Chongqing as a tourist destination.
Code No.	PV
PVQ1	When traveling to Chongqing, the service quality is very good.
PVQ2	When traveling to Chongqing, the management level is very good.
PVQ3	When traveling to Chongqing, I can enjoy delicious food.
PVQ4	When traveling to Chongqing, it is very convenient.
PVQ5	When traveling to Chongqing, the pricing is very reasonable.
PVQ6	When traveling to Chongqing, the cost of living is very low.
PVQ7	When traveling to Chongqing, the cost-effectiveness is very high.
PVQ8	When traveling to Chongqing, it can satisfy my curiosity.
PVQ9	When traveling to Chongqing, I can admire the unique urban scenery.
PVQ10	When traveling to Chongqing, I can experience some special new things.
Code No.	TI
TIQ1	It is very likely that I will choose to travel to Chongqing in the future.
TIQ2	I plan to travel to Chongqing.
TIQ3	I will recommend travel to Chongqing to others.

**Table 2:** Analysis of convergent validity.

Variables	Factor Loadings	Composite Reliability	AVE
SMU	0.677–0.929	0.920	0.661
PGMO	0.832–0.942	0.956	0.812
PA	0.826–0.914	0.953	0.771
PV	0.735–0.887	0.960	0.704
TI	0.907–0.942	0.951	0.865

**Table 3:** Analysis of discriminant validity.

Variables	AVE	1	2	3	4	5
1. SMU	0.661	**0.813**				
2. PGMO	0.812	0.589	**0.901**			
3. PA	0.771	0.492	0.695	**0.917**		
4. PV	0.704	0.466	0.669	0.681	**0.890**	
5. TI	0.865	0.463	0.545	0.689	0.661	**0.930**

Note: The variables’ square roots of AVE are in bold in a diagonal formation.

**Table 4:** Path analysis results.

Hypothesized Paths	Standard Error	Critical Ratio	*p* Value	Standardized Regression Coefficient	Support
SMU → TI	0.038	3.535	0.000	0.126	Yes
SMU → PA	0.041	3.391	0.000	0.122	Yes
SMU → PV	0.037	2.876	0.004	0.106	Yes
PGMO → TI	0.055	−1.531	0.126	−0.078	No
PGMO → PA	0.044	16.453	0.000	0.632	Yes
PGMO → PV	0.040	15.969	0.000	0.616	Yes
PA → TI	0.040	10.721	0.000	0.449	Yes
PV → TI	0.042	9.518	0.000	0.375	Yes

**Table 5:** Analysis of mediating effects.

Indirect Relationships	Point Estimate	Bootstrap 1000 Times	
		Bias-Corrected 95%	
		Lower Bound	Upper Bound
SMU → PA → TI	0.059	0.018	0.126
PGMO → PA → TI	0.309	0.185	0.440
SMU → PV → TI	0.043	0.009	0.102
PGMO → PV → TI	0.252	0.150	0.427

## Data Availability

Datasets analyzed or generated during this study are under the control of the investigators and can be requested via correspondence to the relevant author.
